# Effectiveness of a bioactive food compound in anthropometric measures of individuals with HIV/AIDS: A nonrandomized trial

**DOI:** 10.1371/journal.pone.0191259

**Published:** 2018-02-09

**Authors:** Rosângela dos Santos Ferreira, Rita de Cássia Avellaneda Guimarães, Elenir Rose Jardim Cury Pontes, Lígia Aurélio Bezerra Maranhão Mendonça, Karine de Cássia Freitas, Priscila Aiko Hiane

**Affiliations:** 1 Nutrition Service, University Hospital, Federal University of Mato Grosso do Sul-UFMS, Campo Grande, Mato Grosso do Sul, Brazil; 2 Post Graduate Program in Biotechnology, Catholic University Dom Bosco, Campo Grande, Mato Grosso do Sul, Brazil; 3 Post Graduate Program in Health and Development in the Central-West Region of Brazil, Federal University of Mato Grosso do Sul- UFMS, Campo Grande, Mato Grosso do Sul, Brazil; TNO, NETHERLANDS

## Abstract

**Background:**

Highly Active Antiretroviral therapy (HAART) promotes anthropometric changes in lipid metabolism and glucose in patients with Human Immunodeficiency Virus (HIV). Functional foods play an important role on metabolism. Bioactive Food Compound (BFC) has shown effective results in changes arising from decompensated lipid metabolism due to the effects of HAART on HIV patients. From this perspective, the objective of this study is to evaluate anthropometric indicators and the body composition of patients undergoing HAART before and after consumption of BFC.

**Methods:**

This is a prospective intervention with 180 individuals with HIV undergoing HAART. They formed two groups and were monitored for 3 months: the first group consisted of individuals who consumed BFC (n = 121) at the recommended daily intake of 40 g. The second group consisted of individuals who did not consume BFC (n = 59). We determined body mass index (BMI), waist circumference (WC), waist-hip ratio (WHR), conicity index (CI) and antiretroviral regimen used by the patients.

**Results:**

The BMI among adults (p<0.001), the WC (p<0.001 and p<0.014 for men and women, respectively) and the CI (p = 0.001 and p<0.001 for men and women, respectively) increased at the end of the study in the group of individuals who did not consume BFC and remained stable in the BFC group. There were no changes in WHR in any of the groups evaluated. Regarding the antiretroviral regimens used, we observed that there was no difference between regimens as for BMI, WC, WHR and CI.

**Conclusions:**

The BFC consumed by HIV patients undergoing HAART allowed the maintenance of anthropometric measures without increasing the mean values of conicity index, suggesting that the consumption of this bioactive compound protects the individual against the development of metabolic syndrome (MeS) in patients infected with HIV undergoing antiretroviral therapy.

## Introduction

Advances in highly active antiretroviral therapy (HAART) are highly relevant to increase the survival of patients with Human Immunodeficiency Virus (HIV). However, there are some specificities in the combination of this therapy that promote anthropometric changes in lipid metabolism and glucose [1].

The metabolic syndrome (MeS), present in the HIV population, is related to disorders in lipid and glucose metabolism and central obesity, which may lead to the development of CDs and type 2 diabetes mellitus [2].

Body fat distribution is one of the main triggering factors of metabolic alterations and chronic diseases; therefore, they are determinants for the occurrence of MeS. Thus, the assessment of body composition of individuals with HIV is of great importance in clinical and nutritional practice. Anthropometric indicators are simple, accessible and non-invasive methods that can be used in clinical practice to classify patients as to the risk of diseases related to excess and/or distribution of fat [3,4].

The evaluation of body composition is paramount in the fight against obesity and associated diseases, since it offers subsidies for changes in lifestyle by prescribing diet programs and exercises [5].

In this perspective, functional foods play an important role in lipid metabolism, especially when their components are combined, potentializing their effect when consumed over time [6,7]. In preliminary studies [8–10], a Bioactive Food Compound (BFC) was developed using a combination of functional foods: flaxseed, oat bran and soybean textured protein. Effective results were obtained for the reduction of serum lipids, such as total cholesterol (TC), Low Density Lipoprotein (LDL-C) and triglycerides (TG), in HIV patients with decompensated lipid metabolism due to effects of HAART regimens.

In view of the above, the objective of this study is to evaluate anthropometric indicators of patients undergoing HAART before and after the consumption of BFC to verify whether possible changes in body composition could be related to BFC consumption or to antiretroviral regimens.

## Materials and methods

### Type of study

This is a prospective intervention study with 180 individuals with HIV. undergoing HAART. The subjects were recruited at reference centers for the treatment of HIV/AIDS (Acquired Immunodeficiency Syndrome) in the state of Mato Grosso do Sul (MS), Brazil, between February 2011 and July 2012. The follow-up was carried out for the period of 12 consecutive months, for each participant from their inclusion (“S1 File. Approval letter”).

The eligibility criteria for participants were: having aged 18 years or more, being on HAART using or not lipid-lowering or hypoglycemic medications, perform laboratory tests according to request and control medical participate from beginning to end of study and having signed the term of free and informed consent (“S3 File. Free and Clarified Consent Term”) approved by a Local Ethics Committee. All patients who met these criteria participated in the study (n = 180).

Pregnant women, indigenous, people with opportunistic diseases or mental disabilities, and users of illicit drugs were not included in the study.

All patients received orientations regarding changes in lifestyle (CLS) during monthly ambulatory visits to the dietitian. The CLS consisted of (a) a nutritional guidance on healthy eating and (b) promotion of physical exercises.

**Fig 1:**
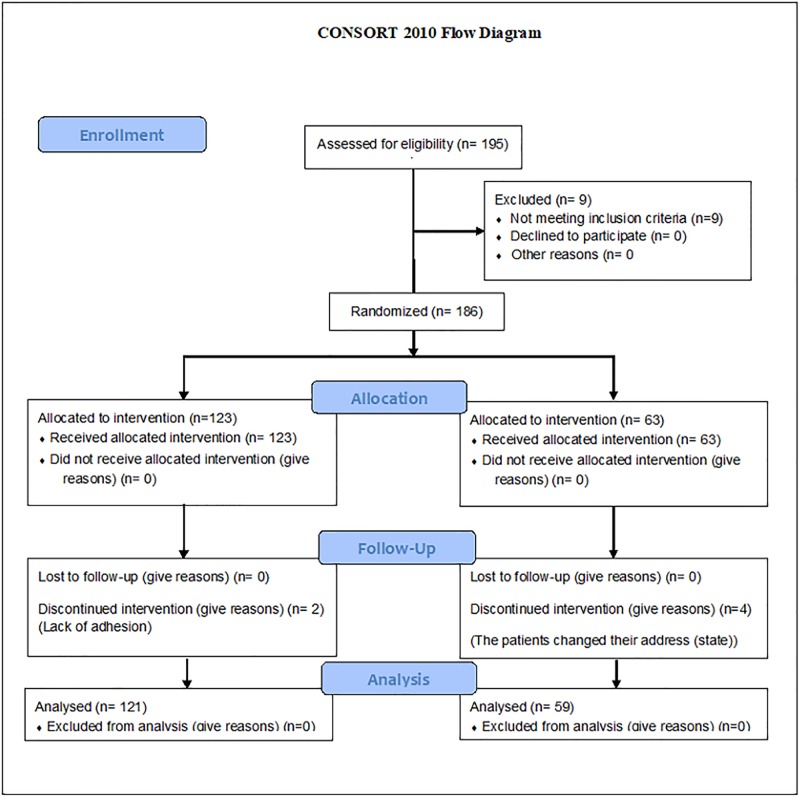
Study design.

For this study, two groups were formed. The first group comprised individuals who agreed to consume BFC (n = 121) and the second group comprised individuals who did not agree to consume BFC (n = 59). For the present study, the first three months of BFC consumption were evaluated. For the first group, the recommended dose of daily BFC consumption was 40 g in sufficient quantities for the study period. The study design is outlined in Fig 1.

The daily dose (40 g) of BFC could be consumed once or fractioned, added in fruit dairy drinks, yogurts, soups and beans, provided it was consumed the same day.

The monthly frequency of nutritional consultations contributed to the adherence of the research participants. Those who consumed the compound had no undesirable effects, which encouraged the continuity of consumption, on the other hand, those who did not consume the compound at their own option, adhered well to the control performed in the clinical follow-up.

### Bioactive food compound (BFC)

The recommended dose of BFC for each subject was 40 g: 20 g of oat bran, 10 g of textured soy protein and 10 g of crushed flaxseed (2:1:1 ratio). The choice of ingredients and their proportions was defined taking into account the presence of functional ingredients in each raw material. The portions were provided in plastic packages protected against light to avoid changes in food composition.

The compound is registered at the National Industrial Property Institute (INPI), Rio de Janeiro (Brazil), under number BR 10 2013 018002 5 (Intellectual Property Agency and Technology Transfer, APITT), as an innovative product developed by the Federal University of Mato Grosso do Sul (UFMS). The record file was published in August 2015 at the Ministry of Development and Foreign Trade, Brazil.

### Study variables

The following study variables were collected: gender, age, anthropometric indicators Body Mass Index (BMI), Waist Circumference (WC), Waist-Hip Ratio (WHR), Conicity Index (CI) and HAART regimen used by patients. All the data of the participants are available in medical individual records in reference centers. The staff member performing the assessments was not involved in any aspect of the intervention and knew the participants only by their study identifier number.

### Body mass index (BMI)

For the nutritional assessment, we used the BMI calculated by weight and height using a Welmy^®^ mechanical scale with a maximum weight of 150 kg and a divided scaling of 0.1 kg, and an aluminum anthropometer coupled to the balance.

For adult individuals (≥ 20 years to < 60 years), the classification was based on WHO (1997) [11]. With regard to elderly (≥ 60 years), the classification of Lipschitz (1994) [12] was used.

**Fig 2:**
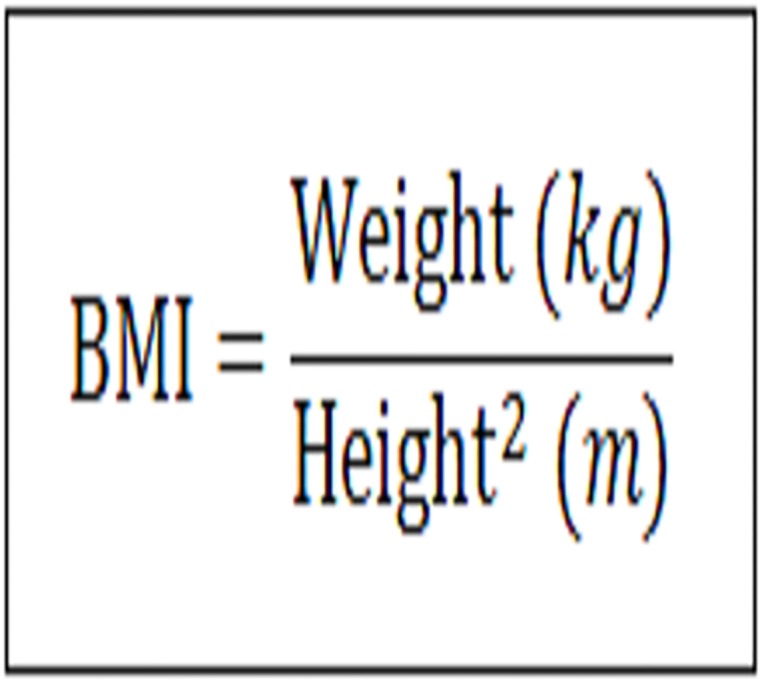
Calculation of body mass index (BMI).

For the calculation of BMI (elderly or adults), the numerical expression was used [13] (Fig 2).

Table 1 shows the classification of the results obtained by BMI.

**Table 1:** Classification of BMI.

Population	BMI Values	Classification
**Adults** ≥ 20 years to < 60 years	≤ 17.9 kg/m^2^	Low weight
	18–24.9 kg/m^2^	Eutrophic
	25–29.9 kg/m^2^	Overweight
	≥ 30 kg/m^2^	Obesity
**Elderly** ≥ 60 years	< 22 kg/m^2^	Slim
	22–27 kg/m^2^	Eutrophic
	> 27 kg/m^2^	Overweight

### Waist circumference (WC)

This measurement reflects the risk of metabolic complications associated with obesity. It is a diagnostic criterion for metabolic syndrome in men and women. The measurement is performed at the largest abdominal perimeter between the last rib and the iliac crest [14,15]. We considered as cut-off points ≥ 102 cm for men and ≥ 88 cm for women [16].

### Waist-hip ratio (WHR)

WHR is a criterion that characterizes the metabolic syndrome (cut-off values of 0.90 cm for men and 0.85 cm for women). It is determined by dividing the perimeter of the waist by the perimeter of the hip [14].

### Conicity index (CI)

**Fig 3:**
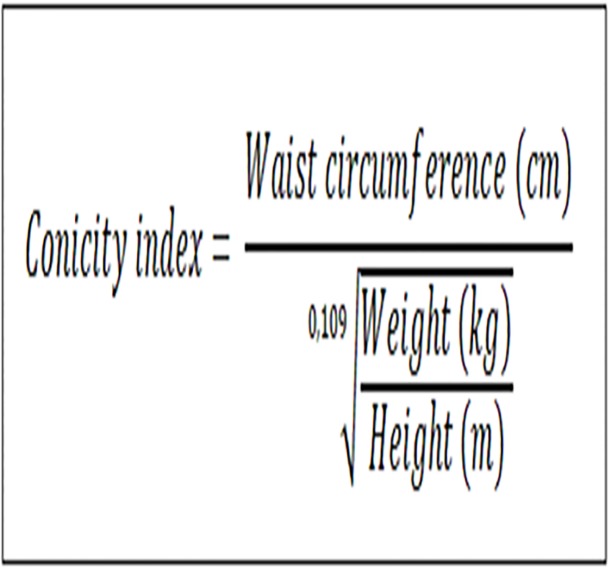
Conicity index (CI).

For the CI, waist and height measurements were taken into account. The mathematical equation was used [17] (Fig 3):

Variable considered as presence of coronary risk for women who obtained a score > 1.18, and for men a score > 1.25 [18].

The anthropometric variables BMI, WC, WHR and CI were measured using a Flexible metal anthropometric tape Sanny^®^ subdivided into 0.1 cm and an anthropometric scale coupled with a Welmy^®^ stadiometer.

### HAART cassification

The HAART regimen was classified into the following groups (Table 2).

**Table 2:** Classification of antiretroviral regimen.

Groups	HAART regimen
**I**	2 NTRI + 1 PI or 2 NTRI + 1 NNTRI + PI
**II**	2 NTRI + 2 PI (with ritonavir) or 2 NTRI + 1 NNTRI + 2 PI
**III**	2 NTRI + 1 NNTRI

NTRI = Nucleoside analog reverse-transcriptase inhibitors. NNTRI = Non-nucleoside reverse-transcriptase inhibitors. PI = protease inhibitors.

### Statistical analysis

The measurements of anthropometric variables were calculated before the beginning of the treatment with BFC and 3 months thereafter in the NO BFC and the BFC groups.

To compare differences (after 3 months minus baseline values) between measurements of the HAART regimen (groups I, II and III), the Kruskal Wallis test, followed by Dunn test, was used (CV > 70%). To compare differences (after 3 months minus baseline values) between the measurements of the groups (BFC and NO BFC), the Mann Whitney test was used (CV > 70%). A multiple linear regression was performed to verify possible confounding factors.

To compare the measurements of each group (after 3 months, baseline values), Student t test using paired samples was performed after verifying the normal distribution by Coefficient of Variation (CV < 20%). A significance level of 5% was adopted.

### Ethical considerations

The ethical approval was granted by the Local Ethics Committee (Federal University of Mato Grosso do Sul) under protocol number 1630 on October 29th, 2009 (“S6 File. Research Project”). An informed consent was signed by all individuals. The authors confirm that all ongoing and related trials for this intervention are registered under number U1111-1199-3532 (Brazilian Registry of Clinical Trials—Ministry of Health, Brazil) (“S7 File. Trendstatement_TREND_Checklist”, “S8 File. Brazilian Registry of Clinical Trials”).

The registration of this clinical trial occurred after its approval by the Local Ethics Committee due to the lack of the database of the Brazilian Registry of Clinics at that time, which justified the later recruitment of participants.

The authors state that all ongoing trials and also related trials of this intervention are recorded.

### Study protocol

In relation to the study protocol (“S9 File. Database”), there were few changes: 20 more participants in the expected sample amount and inclusion of one more study variable, the Conicity Index. Regarding the bromatological, microbiological and sensory analysis of the BFC and the lipid profile after consumption have already been published in other manuscripts [9, 10].

## Results

According to Table 3, upon analyzing the anthropometric measures at the beginning of the study and after 3 months, there were no differences between HAART regimens I, II and III in the group that consumed BFC. However, there was a difference in the NO BFC group for the variables BMI, WC and CI. There was a difference between those who consumed BFC and those who did not, except for BMI in HAART I and for the anthropometric measure WHR.

**Table 3:** Differences (after 3 months minus baseline) in the values of anthropometrical variables in the groups BFC/NO BFC according to HAART regimen.

Variables	HAART	BFC group	NO BFC group	*p*^(1)^
		Mean ± SD		
**BMI, kg/m**^**2**^	**I**	0.376 ± 1.450	^a^ 1.195 ± 1.240	0.077
	**II**	- 0.158 ± 1.239	^b^ 0.798 ± 0.991	**<0.001**
	**III**	- 0.227 ± 1.087	^a^ 1.480 ± 1.055	**<0.001**
***p***^(2)^		0.692	**0,044**	
**WC, cm**	**I**	0.880 ± 3.879	^a^ 5.850 ± 4.259	**0.040**
	**II**	-0.225 ± 3.680	^b^ 2.597 ± 4.360	**<0.001**
	**III**	-0.339 ± 4.107	^a^ 8.975 ± 16.454	**<0.001**
***p***^(2)^		0.815	**0**.**009**	
**WHR, cm**	**I**	-0.005 ± 0.020	0.003 ± 0.005	0.525
	**II**	0.010 ± 0.087	0.005 ± 0.019	0.361
	**III**	0.004 ± 0.049	0.010 ± 0.036	0.706
***p***^(2)^		0.913	0.665	
**CI**	**I**	0.012 ± 0.026	^a^ 0.123 ± 0.134	**0.011**
	**II**	0.004 ± 0.035	^b^ 0.024 ± 0.045	**0.005**
	**III**	0.011 ± 0.060	^b^ 0.052 ± 0.052	**<0.001**
***p***^(2)^		0.811	**0.048**	

BFC = Bioactive Food Compound (BFC). SD = standard deviation. BMI = Body Mass Index. WHR = Waist-hip ratio. WC = Waist circumference. CI = Conicity Index. Antiretroviral Regimen I = 2 NTRI + 1 PI or 2 NTRI + 1 NNTRI + PI; II = 2 NTRI + 2 PI (with ritonavir) or 2NTRI + 1 NNTRI + 2 PI; III = 2 NTRI + 1 NNTRI. *P* values in bold indicate a statistically significant difference (*p* ≤ 0.05).

^(1)^ Mann Whitney test.

^(2)^ Kruskal Wallis test followed by Dunn test (different letters indicate statistically significant differences).

There were no differences only for the anthropometric indicator WHR in groups that consumed BFC or not. Therefore, it can be inferred that the worsening of the anthropometric profile is due to the non-consumption of the compound, and not because of the type of HAART regimen (Table 3).

Another observation reinforcing the statement that changes in anthropometric measures were due to BFC consumption is that, for some variables, a satisfactory result concerning the reduction of a certain anthropometric parameter related to the HAART regimen, reported for the group that consumed BFC, contrasted with a worse result for the group that did not consume BFC. As an example, we may mention that the greatest reduction in BMI values occurred in the group treated with the HAART III regimen. In contrast, there was an increase in the BMI of the group that did not consume BFC regarding this same HAART regimen. This was also observed with respect to WC (Table 3).

According to the multivariate analysis (Table 4), there was an association between individuals who consumed BFC and those who did not, and the changes were observed in anthropometric measurements. There was no association with the following variables: age, gender and type of HAART.

**Table 4:** Results of the multiple linear regression.

Variables	Differences (after 3 months minus baseline values)			
	BMI, kg/m^2^	WC, cm	WHR, cm	CI
BFC	**p < 0.001**	**p < 0.001**	p = 0.998	**p < 0.001**
HAART	p = 0.955	p = 0.289	p = 0.782	p = 0.986
Age	p = 0.504	p = 0.240	p = 0.718	p = 0.512
Gender	p = 0.367	p = 0.320	p = 0.543	p = 0.996

BMI = Body Mass Index. WHR = Waist-hip ratio. WC = Waist circumference. CI = Conicity Index. BFC = Bioactive Food Compound. HAART = Antiretroviral Regimen (I = 2 NTRI + 1 PI or 2 NTRI + 1 NNTRI + PI; II = 2 NTRI + 2 PI (with ritonavir) or 2NTRI + 1 NNTRI + 2 PI; III = 2 NTRI + 1 NNTRI). *P* values in bold indicate a statistically significant difference (*p* ≤ 0.05).

Based on multivariate results, there was no need to stratify the values of anthropometric measures by age, gender and HAART. Therefore, Table 5 shows the mean values of the anthropometric measurements according to age and BMI, and according to gender in the other measurements since there were different parameters (range of values considered normal, as described in the Materials and methods section).

**Table 5:** Anthropometrical variables of the BFC/NO BFC groups.

Variables	n	BFC group		n	NO BFC group	
		Mean ± SD *p*			Mean ± SD *p*	
		Baseline	After		Baseline	After
BMI, Kg/m^2^						
Adults	102	25.51 ± 4.54	25.39 ± 4.44	53	27.51 ± 5.16	28.68 ± 5.14
		0.309			**<0.001**	
Elderly	19	25.63 ± 4.13	25.40 ± 4.02	6	24.49 ± 2.67	24.95 ± 2.83
		0.395			0.122	
WC, cm						
Females	64	95.00 ± 9.70	94.56 ± 9.43	28	100.71±15.69	104.41 ± 14.69
		0.369			**<0.001**	
Males	57	95.69 ± 11.70	95.81 ± 12.37	31	98.73 ± 10.53	105.69 ± 20.09
		0.817			**0.014**	
WHR, cm						
Females	64	0.94 ± 0.07	0.94 ± 0.10	28	0.99 ± 0.17	0.99 ± 0.17
		0.546			0.188	
Males	57	0.97 ± 0.07	0.97 ± 0.09	31	0.97 ± 0.07	0.98 ± 0.08
		0.474			0.152	
CI						
Females	64	1.34 ± 0.07	1.34 ± 0.07	28	1.36 ± 0.09	1.41 ± 0.11
		0.794			**0.001**	
Males	57	1.33 ± 0.08	1.34 ± 0.08	31	1.32 ± 0.08	1.36 ± 0.09
		**0.046**			**<0.001**	

BFC = Bioactive Food Compound (BFC). SD = standard deviation. BMI = Body Mass Index. WHR = Waist-hip ratio. WC = Waist circumference. CI = Conicity Index. Student *t* Test using paired samples (baseline x after 3 months). The *p* values in bold indicate a statistically significant difference (*p* ≤ 0.05).

Adults in the group that consumed BFC had BMI values close to 25 kg/m^2^, that is, the lowest limit of the range corresponding to overweight (25–29.9 kg/m^2^). They remained in this condition 3 months after the consumption of BFC without differences in the average BMI. On the other hand, adults who did not consume BFC had an increase in the mean BMI towards the upper limit of the range corresponding to overweight (from 27.51 ± 5.16 to 28.68 ± 5.14 kg/m^2^). The elderly remained eutrophic in both groups (BFC and NO BFC).

Women presented values for WC above the cut-off point of 88 cm. However, there was increase in this measurement 3 months after the monitoring in the NO BFC group (from 100.71 ± 15.69 cm to 104.41 ± 14.69 cm). For men, there was also an increase in WC in the NO BFC group (from 98.73 ± 10.53 cm to 105.69 ± 20.09 cm). These parameters are above the cut-off point of 102 cm.

Regarding the measurements taken at the beginning and at the end of the study, we verified that, in relation to the WHR, there were no significant differences, although there was an increase in BMI, WC and CI in the group that did not consume BFC. The values obtained for WHR were higher than the cut-off point of 0.90 cm for men and 0.85 cm for women.

Patients presented mean values of CI above the cut-off point (> 1.18 for women and > 1.25 for men). There was no increase in CI in women who consumed BFC, but there was an increase in women who did not consume it (from 1.36 ± 0.09 to 1.41 ± 0.11). For men, there was an increase in both groups, BFC and NO BFC. However, such an increase was observed for the group that did not consume BFC (from 1.32 ± 0.08 to 1.36 ± 0.09), when compared to the group that consumed BFC (from 1.33 ± 0.08 to 1.34 ± 0.08).

## Discussion

As for anthropometric factors, we observed an increase in BMI values in adults and increases in WC and CI in the group of patients who did not consume BFC. All the changes we observed in anthropometric measurements are because the “NO BFC group” did not consume BFC, and did not relate to the type of HAART regimen.

It is known that the treatment with long-term HAART causes metabolic abnormalities (dyslipidemia, insulin resistance and changes in body fat), predisposing to the occurrence of MeS, which is strongly characterized by an aggregation of central obesity, an increased risk of coronary heart disease (CD) and type 2 diabetes mellitus (DM 2) [19–22].

Because MeS is associated with the development of DM 2 and increased risk of CD, it is necessary to know the magnitude of cardiovascular risk in the population with HIV/AIDS undergoing HAART [23] and to develop strategies for protection, especially in Brazil, where it was established from 2013 that all virus-infected individuals should initiate antiretroviral treatment as soon as possible. This predisposes them to the development of chronic noncommunicable diseases (CNCD) [24].

In vitro studies have evidenced an inhibitory effect of PIs on GLUT4, which causes insulin resistance in HIV-positive individuals [25,26] and affects the transcription factor SREBP-1 (steroid regulatory element binding protein-1c), which in turn affects the glucose metabolism by producing imperfect peroxisome proliferator- activated gamma receptor (PPAR-y), which plays an important role in the metabolism of glucose and lipids [27].

There is also a strong correlation between BMI and waist circumference in HIV-positive patients taking protease inhibitors. This evidences that the higher the BMI, the greater the waist circumference. Consequently, this contributes to the onset of chronic diseases, including systemic arterial hypertension [28].

The combination of HAART regimen with NRTIs, NNRTIs and PIs increases the risk of developing morphological changes, such as severe lipodystrophy [29]. There are studies associating worse results of metabolic changes with body fat accumulation and PI-based therapy [30–32]. It is noteworthy to mention that, in this study, there were no relations between changes in anthropometric indicators and antiretroviral regimens using PI.

When considering that the choice for the type of antiretroviral therapy depends on the clinical condition of the patient and that metabolic changes are inherent to the use of such medication, regardless of the type of therapy, a dietary adjustment becomes essential to help, in a supportive way, to reduce the imminent risk of obesity, which is a component of MeS.

The BFC used in this study was effective in adapting body composition since BFC components are associated with the control of anthropometric parameters. Studies report that crushed flax, when consumed regularly, proved to be effective in significantly reducing BMI, WC and hip circumference (HC) due to lignans [33].

This is also true for WC when dietary fibers of oat cereal are consumed for 6 weeks [34], and also for components of soybeans, which are related to the reduction of CD risk factors [35,36].

In this sense, as soon as antiretroviral therapy begins, a nutritional monitoring should also begin, including functional feeding, monitoring of anthropometric measurements and monitoring of lipid profile in clinical practice through routine laboratory exams.

There is evidence that anthropometric indicators of centralized obesity are able to predict co-morbidities and mortality in addition to establishing criteria for an indirect assessment of the risk of CD and SM [37–39].

BMI, widely used in clinical practice because it is easy to be determined in adults and in elderly, has limitations as to the identification of body composition and may provide false diagnoses of overweight [40].

WC is an important indicator of MeS. In this study, both genders presented values of abdominal circumference above the cut-off point, evidencing MeS with risk of CD. However, there is a weak correlation between anthropometric indexes and cardiovascular risk factors. However, the measurement of WC in overweight/obese men correlated more strongly with MeS [41]. This is another index that provides satisfactory and similar performances to discriminate high coronary risks [42].

The relation between waist and hip, in this study, was not related to BFC consumption. WHR, previously used as a cardiovascular risk factor, is no longer used, since the measurement of WC has shown to provide a more reliable correlation [40].

Regarding CI, in this study, we observed mean values above the cut-off point for both genders. CI is another anthropometric indicator considered as predictor of relevant events such as MeS and risk for CD [43–46].

Studies on metabolic changes and anthropometric indicators (weight, height, WC, thigh circumference, BMI, adiposity index, WHR) have shown that, among the most diverse anthropometric indicators, WC is convenient, low-cost and reliable to indicate a better performance in the identification of MeS in the males and females, besides being considered a good tool to separately identify each metabolic disorder taking into account the biochemical parameters of the lipid and glycemic profiles. Therefore, it is recommended to use it in routine clinical practice to prevent cardiovascular complications in patients with HIV [1].

Therefore, the consumption of BFC by patients undergoing antiretroviral therapy allowed the maintenance of anthropometric measures within a normal range, protecting them against overweight, without increasing the mean values of CI.

Previous results indicate that BFC exerts an antiatherogenic effect on glycemic control and reduces triglycerides [9, 10], which is probably due to its chemical composition, its high linolenic acid, soluble fibers and isoflavone contents, and the benefits observed in this study.

Therefore, we conclude that the consumption of this bioactive compound may be a preventive measure against the development of MeS in HIV-infected patients regardless of the type of antiretroviral therapy.

## Supporting information

S1 File Approval letter. (PDF)

S2 File Approval letter in portuguese. (PDF)

S3 File Free and Clarified Consent Term. (PDF)

S4 File Free and Clarified Consent Term in portuguese. (PDF)

S5 File Research project in Portuguese. (PDF)

S6 File Research project. (PDF)

S7 File Trendstatement_TREND_Checklist. (PDF)

S8 File Brazilian Registry of Clinical Trials. (PDF)

S9 File Database. (XLSB)
