# Supplementary material for: Effectiveness of a bioactive food compound in anthropometric measures of individuals with HIV/AIDS: A nonrandomized trial
Source: PLoS One. 2018 Feb 9;13(2):e0191259. doi: 10.1371/journal.pone.0191259 (PMC5806863; doi:10.1371/journal.pone.0191259)
Supplement: S1 File — (PDF) [file pone.0191259.s001.pdf]

Approval letter

My signature in this document states protocol n. 1630 of the Researcher Rosângela dos Santos Ferreira entitled "**Bioactive Compound: Nutritional Therapeutics in the lipid and glycemic alterations by HIV infection in individuals using Combined Antiretroviral Therapy**" and its Free And Clarified Consent Term were reviewed by this committee and approved at a regular meeting on October 29, 2009 in accordance with the normative resolutions of the Ministry of Health.
