# Supplementary material for: Effectiveness of a bioactive food compound in anthropometric measures of individuals with HIV/AIDS: A nonrandomized trial
Source: PLoS One. 2018 Feb 9;13(2):e0191259. doi: 10.1371/journal.pone.0191259 (PMC5806863; doi:10.1371/journal.pone.0191259)
Supplement: S2 File — (PDF) [file pone.0191259.s002.pdf]

ANEXO - Carta de aprovação do Comitê de Ética em Pesquisa/UFMS.

Universidade Federal de Mato Grosso do Sul
Comitê de Ética em Pesquisa /CEP/UFMS

Carta de Aprovação

*A minha assinatura neste documento, atesta que o protocolo nº 1630 da Pesquisadora Rosângela dos Santos Ferreira intitulado "**Composto Bioativo: Terapêutica Nutricional nas alterações lipídicas e glicêmicas pela infecção do HIV em indivíduos em uso de Terapia Antiretroviral combinada**" e o seu Termo de Consentimento Livre e Esclarecido, foram revisados por este comitê e aprovados em reunião ordinária no dia 29 de outubro de 2009, encontrando-se de acordo com as resoluções normativas do Ministério da Saúde.*

Prof. Paulo Roberto Haidamus de Oliveira Bastos
Coordenador em exercício do Comitê de Ética em Pesquisa da UFMS

Campo Grande, 29 de outubro de 2009.

Comitê de Ética da Universidade Federal de Mato Grosso do Sul
http://www.propp.ufms.br/bioetica/cep/
bioetica@propp.ufms.br
fone 0XX67 345-7187
