# Supplementary material for: Effectiveness of a bioactive food compound in anthropometric measures of individuals with HIV/AIDS: A nonrandomized trial
Source: PLoS One. 2018 Feb 9;13(2):e0191259. doi: 10.1371/journal.pone.0191259 (PMC5806863; doi:10.1371/journal.pone.0191259)
Supplement: S3 File — (PDF) [file pone.0191259.s003.pdf]

FREE AND CLARIFIED CONSENT TERM

RESEARCH NAME: BIOATIVE COMPOUND: NUTRITIONAL THERAPEUTICS IN LIPIDIC AND GLYCEMIC ALTERATIONS BY HIV INFECTION IN INDIVIDUALS USING COMBINED ANTIRETROVIAL THERAPY.

Research Coordinator: Rosângela dos Santos Ferreira

INFORMATION and purpose of research: 1- Procedures used and purpose; 2 - Discounts and expected risks; 3 – possible benefits.

We are developing a research to understand the effects of bioactive food consumption on lipid and blood glucose alterations in HIV/AIDS infected individuals attended at the Hospital Dia Profº Esterina Corsini - Maria Pedrossian University Hospital (HUMAP), Universidade Federal de Mato Grosso do Sul and Hospital Dia at the Infectious-Parasitic Diseases Center (CEDIP) of the Municipal Secretariat of Public Health - Campo Grande, MS.

The present project aims at evaluating the influence of nutritional intervention in the development of dyslipidemia and hyperglycemia in HIV positive individuals who use antiretroviral therapy (ART) by means of food compound with functional activity.

Patients will be submitted to nutrition consultation, using clinical resources such as: **anthropometry**, aiming at measuring body weight, height, abdomen circumference, waist-hip circumference; **Food consumption**, through the retrospective method of 24-hour recall, food frequency questionnaire and dietary history. All patients will receive guidance on healthy eating, low in fat and sugars. Some patients will be randomly selected to receive all procedures from conventional nutrition consultation described above and food compound containing oat bran, flaxseed and textured soy protein for consumption for 3 months.

Laboratory exams routinely requested by doctors every 3 months will be attached to the medical record. Results will be transcribed into a structured research form, which will be analyzed and evaluated by the nutritionist coordinating the study.

At the end of the research, it is expected that consumption of bioactive food compound has interfered positively in the control and reduction of total cholesterol, triglycerides, glucose and insulin.

This research does not expose the participant to any life risk and participant may desist from the study at any time.

Therefore, ____ (name of participant), RG______________________ have received above information. I am aware of my rights listed below and I agree to participate in the survey.

1 - Guarantee to receive answer to any question or clarification of doubt about procedures, risks, benefits and other subjects related to the research;

2 - Freedom to withdraw consent at any time and to stop participating in the study without this causing damage to the continuation of my care and nutritional treatment;

3 - Security of not being identified and maintenance of confidentiality of information related to privacy;

Campo Grande/MS,____________(month) __________(day)___________(year)

____

Participant signature

To contact research coordinator: Rosângela dos Santos Ferreira, nutritionist at the Division of Nutrition and Dietetics/Nucleus of University Hospital of the Federal University of Mato Grosso do Sul. Avenida Felinto Müller, s / nº 79063-090. Tel: (67) 3345 3048/3345 3037.

Telephone of the Research Ethics Committee on Human Beings (REC) / UFMS (67) 3345 7187
