# Supplementary material for: Effectiveness of a bioactive food compound in anthropometric measures of individuals with HIV/AIDS: A nonrandomized trial
Source: PLoS One. 2018 Feb 9;13(2):e0191259. doi: 10.1371/journal.pone.0191259 (PMC5806863; doi:10.1371/journal.pone.0191259)
Supplement: S4 File — (PDF) [file pone.0191259.s004.pdf]

TERMO DE CONSENTIMENTO LIVRE E ESCLARECIDO

NOME DA PESQUISA: COMPOSTO BIOATIVO: TERAPÊUTICA NUTRICIONAL NAS ALTERAÇÕES LIPÍDICAS E GLICÊMICAS PELA INFECÇÃO DO HIV EM INDIVÍDUOS EM USO DE TERAPIA ANTIRETROVIAL COMBINADA.

Coordenadora da pesquisa: Rosângela dos Santos Ferreira

INFORMAÇÕES e objetivo da pesquisa: 1- Os procedimentos que serão utilizados e seu propósito; 2 – Os descontos e riscos esperados; 3 – Os benefícios que podem obter.

Estamos desenvolvendo uma pesquisa para conhecer os efeitos do consumo de alimentos com função bioativa nas alterações dos lipídeos e glicose sanguínea em indivíduos infectados pelo HIV/AIDS atendidos no Hospital Dia Profº Esterina Corsini do Hospital Universitário Maria Pedrossian (HUMAP) da Universidade Federal de Mato Grosso do Sul e no Hospital Dia do Centro de doenças Infecto-Parasitárias (CEDIP) da Secretaria Municipal de Saúde Pública – Campo Grande, MS.

O presente projeto tem o objetivo de avaliar a influência da intervenção nutricional no desenvolvimento de dislipidemia e hiperglicemia em indivíduos HIV positivo que utilizam terapia antirretroviral (TARV) por meio de composto alimentar com atividade funcional.

Os pacientes serão submetidos à consulta de nutrição, utilizando recursos clínicos como: **antropometria**, visando aferir o peso corporal, estatura, circunferência do abdômen, contura-quadril; **consumo alimentar,** através do método retrospectico de recordatório de 24 horas, questionário de frequência alimentar e história dietética. Todos os pacientes receberão orientação sobre alimentação saudável, pobre em gorduras e em açúcares. Alguns pacientes serão selecionados aleatoriamente para além de receberem todos os procedimentos provenientes da consulta de nutrição convencional, como descrito acima, receberão um composto alimentar contendo farelo de aveia, linhaça e proteína texturizada de soja para consumo durante 3 meses.

Por meio de investigação em prontuário dos resultados dos exames laboratoriais solicitados, de 3 em 3 meses rotineiramente, pelos médicos e anexados em prontuário, será transcrito para o questionário estruturado da pesquisa, para análise e avaliação dos resultados.

Ao final da pesquisa, espera-se evidenciar que o consumo do composto alimentar bioativo interferiu positivamente no controle e redução do colesterol total, triglicerídeos, glicose e insulina.

Essa pesquisa não expõe o participante a qualquer risco de vida e em qualquer momento poderá desistir do estudo.

Sendo assim, eu ____(nome do participante), RG______________________, tendo recebido as informações acima, e ciente dos meus direitos abaixo relacionados, concordo em participar da pesquisa.

1 – Garantia de receber resposta a qualquer pergunta ou esclarecimento de dúvida acerca dos procedimentos, riscos, benefícios e outros assuntos relacionados com a pesquisa;

2 – Liberdade de retirar o consentimento a qualquer momento e deixar de participar do estudo sem que isso traga prejuízo a continuação do meu cuidado e tratamento nutricional;

3 – Segurança de que não ser identificado e manutenção do caráter confidencial das informações relacionadas com privacidade;

Campo Grande/MS, __________(dia)____________(mês)___________(ano)

____

Assinatura do participante

Para contato com a coordenadora da pesquisa: Rosângela dos Santos Ferreira, nutricionista lotada na Divisão de Nutrição e Dietética/ Núcleo de Hospital Universitário da Universidade Federal de Mato Grosso do Sul. Avenida Felinto Müller, s/nº 79063-090. Tel: (67) 3345 3048 / 3345 3037.

Telefone do Comitê de Ética em Pesquisa co Seres Humanos (CEP)/ UFMS (67) 3345 7187.
