# Supplementary material for: Effectiveness of a bioactive food compound in anthropometric measures of individuals with HIV/AIDS: A nonrandomized trial
Source: PLoS One. 2018 Feb 9;13(2):e0191259. doi: 10.1371/journal.pone.0191259 (PMC5806863; doi:10.1371/journal.pone.0191259)
Supplement: S5 File — (PDF) [file pone.0191259.s005.pdf]

COMPOSTO BIOATIVO: TERAPÊUTICA NUTRICIONAL NAS ALTERAÇÕES LIPÍDICAS E GLICÊMICAS PELA INFECÇÃO DO HIV EM INDIVÍDUOS EM USO DE TERAPIA ANTIRETROVIRAL COMBINADA.

ROSÂNGELA DOS SANTOS FERREIRA

E-mail:rosangela.ferreira@ufms.br

Coordenadora

Universidade Federal de Mato Grosso do Sul

2009

Campo Grande-MS

1. REVISÃO DA LITERATURA

Na América Latina, o Brasil foi o primeiro país a adotar a política de distribuição universal da terapia medicamentosa para a população infectada pelo HIV/Aids. Desde 1991, as drogas antiretrovirais foram distribuídas de forma universal e, a partir de 1996, após a XI Conferência Internacional de Aids, realizada em Vancouver, houve um marco histórico, onde o programa brasileiro passou a ter a política de distribuição da terapia antiretroviral (TARV) de forma gratuita, obrigatória e universal, garantida pela lei nº 9.313 de 13/11/1996(1).

A partir daí, houve uma mudança progressiva do perfil de morbi-mortalidade da infecção pelo HIV, demonstrado através de redução das internações hospitalares, das ocorrências de infecções oportunistas associadas e, conseqüentemente dos óbitos secundários a Aids. A doença passou a ter um melhor prognóstico; a sobrevida dos pacientes aumentou, a infecção pelo HIV passou a ser considerada como uma doença de caráter evolutivo e crônico e potencialmente controlável (2,3).

A TARV de alta potência está na combinação de três classes de drogas antiretrovirais, associada a dois Inibidores da Transcriptase Reversa Analágos Nucleosídeos (ITRNs) com um Inibidor da Protease (IP) ou com um Inibidor da Transcripase Reversa Não Nucleosídeo (ITRNN), demonstrando controle na evolução da infecção, com melhor prognóstico de vida desses indivíduos (4). Por outro lado, eventos adversos associados à terapêutica estão sendo evidenciadas pelo desenvolvimento da síndrome de lipodistrofia, caracterizada por dislipidemia, ocorrendo hipercolesterolemia e hipertrigliceridemia, alterações glicêmicas e alterações morfológicas com lipoatrofia e lipohipertrofia (5).

Estudos mostram os efeitos colaterais dos medicamentos em alterações lipídicas e glicêmicas. No estudo de Domingues (4), dentre os IP, observou-se hipercolesterolemia

em 68,0%, 57,1%, 45,6% e 32,6% em pacientes em uso de indinavir/ritonavir, de saquinavir/ritonavir, de nelfinavir e de lopinavir/ritonavir, respectivamente.

Nesse cenário, a intervenção nutricional tem tido um impacto positivo na prevenção e controle primário das alterações do perfil lipídico e glicêmico dos indivíduos (6,7).

Buscando reunir estratégias alimentares com vista ao tratamento coadjuvante para os efeitos colaterais advindos da TARV de alta potência, propõe-se a utilização de alimentos com fins especiais, como se apresentam os alimentos funcionais pelas suas propriedades bioativas, além das nutricionais básicas; sendo consumidos em dietas convencionais da alimentação cotidiana, demonstrando capacidade de regular funções corporais e trazendo benefícios fisiológicos específicos; de forma a auxiliar na proteção contra doenças como doenças cardiovasculares (DAC) e diabetes mellitus (8,9). Dessa maneira, reforça-se o interesse em estudar esse protocolo alimentar por meio de um composto bioativo em indivíduos infectados pelo HIV.

Pretende-se, então, introduzir alimentos funcionais como parte de alimentação saudável nesses indivíduos, com vistas ao controle e terapêutica coadjuvante no desenvolvimento de DAC e na alteração glicêmica decorrentes do uso de TARV.

Este estudo tratará do desenvolvimento de um composto bioativo que será constituído por: **semente de linhaça** *(Linum usitatissimum)* contém substâncias como a lignana, as fibras e o ácido graxo linolênico as quais apresentam efeitos cardioprotetores (10), com dosagem terapêutica de 10 g (01 colher de sobremesa), adequada para o modelo da aterosclerose, apropriada para atuar como preventivo e terapêutico na regressão da aterosclerose e de conseqüentes doenças cardiovasculares; **aveia** *(Avena sativa)* na forma de apresentação em flocos grossos, representado pelo farelo e aveia, por possuir maior fonte dietética de B-glucana, na quantidade de 20 g (1 colher de sopa), própria para manutenção e ajuste de glicemia dentro dos níveis de normalidade; **soja**

texturizada *(Glycine Max)* na dosagem de 10 g (02 colheres de sopa), proteína com baixo teor de lipídeo saturado e colesterol em sua composição química, com propriedade hipocolesterolêmica, de diminuição da agregação plaquetária e das lesões ateroscleróticas. Esses alimentos apresentam efeitos cardioprotetores e adequação glicêmica (11).

As quantidades estabelecidas serão consideradas, neste estudo, como dosagens teste para os fins propostos, quais sejam: redução do colesterol total (CT), da lipoproteína de alta densidade (LDL-c), dos triglicerídeos (TG) e da glicose sangüinea.

Palavras-chave: HIV, Aids , dislipidemias, hiperglicemia, alimentos funcionais.

2. ANTECEDENTES E JUSTIFICATIVA

O Brasil vem passando por um processo de inversão das curvas de mortalidade, denominado transição epidemiológica, em que se observa um declínio na mortalidade por doenças infecciosas e um concomitante aumento na mortalidade por doenças crônicas não transmissíveis (2).

Atualmente a epidemia de HIV/Aids no país é considerada estável, assumindo um caráter de doença crônica, devido a terapia antiretroviral de alta potência (TARV). Estudos apontam que cerca de dois mil adultos diagnosticados com HIV/ Aids, entre 1998 e 1999, mais da metade deles (60%) continuaram vivos por, no mínimo, 108 meses depois do diagnóstico, confirmando o crescimento na sobrevida dos pacientes brasileiros. Uma série histórica de 1980 a junho de 2008, foi registrado 506.499 casos de aids no Brasil. Durante esses anos, 205.409 mortes ocorreram em decorrência doença (2,3).

A região Sudeste é a que tem o maior percentual de notificações, 60,4% ou seja, 305.725 casos. O Sul concentra 95.552 (18,9%), o Nordeste 58.3481 (15%), o Centro-Oeste 28.719 (5,7%) e o Norte 18.155 (3,6%) (13).

O diagnóstico precoce, seguido do acesso a medicamentos antiretrovirais e do acompanhamento clínico adequado contribuem para aumentar a sobrevida desses pacientes. O uso de TARV tem impacto positivo no tempo de vida após o diagnóstico; em pacientes diagnosticados ainda em fase assintomática, tendo maior sobrevida que aqueles que já desenvolveram doenças oportunistas. Pacientes de maior escolaridade (níveis médio e superior) vivem mais; mulheres têm maior sobrevida; os infectados por via sexual vivem mais que os expostos ao vírus por uso de drogas injetáveis; aqueles que fazem profilaxia de pneumocistose (pneumonia potencialmente grave) também vivem mais tempo (1).

Os países como o Brasil, que optaram pelo acesso universal ao tratamento na década de 1990, determinaram a mudança na história natural da doença. Além da oferta universal da TARV de alta potência, a estruturação do sistema de saúde foi fundamental para melhorar a qualidade de vida das pessoas que vivem com HIV/Aids (1). Porém, ainda é preciso fortalecer a resposta dos serviços de saúde aos efeitos adversos do tratamento, por meio de incentivo a um estilo de vida saudável (alimentação adequada e atividade física), prevenindo a ocorrência de doenças cardiovasculares e da lipodistrofia (6).

A nutrição clínica funcional é uma forma contemporânea de abordar a ciência da nutrição, tendo como propósito avaliar a interação do organismo com o alimento e o processo da nutrição. Dessa maneira, os alimentos funcionais são capazes de atuar no metabolismo e na fisiologia humana, promovendo efeitos benéficos à saúde, podendo retardar o estabelecimento de doenças crônicas e/ou degenerativas e melhorar a qualidade e a expectativa de vida das pessoas. A nutrição funcional leva em

consideração a importância da integridade fisiológica e funcional do trato gastrintestinal (9).

Torna-se importante, então, interferir nos hábitos e processos alimentares para efetivamente interferir nos efeitos colaterais advindos da TARV.

Com base neste contexto, surge a nutrição funcional com alguns princípios que possibilitam assegurar melhor oferta de substratos ao organismo, determinando uma nova era no controle das alterações lipídicas e glicêmicas dos indivíduos submetidos à TARV.

Justifica-se, então a busca de estratégias nutricionais que visem prevenir e participar do controle das adversidades decorrentes dos antiretrovirais nos indivíduos infectados pelo HIV/Aids.

3. METODOLOGIA

Este estudo foi idealizado pela seguinte hipótese: "Há relação entre uma dieta com atividade funcional (composto bioativo), dislipidemias, alteração glicêmica em indivíduos infectados pelo HIV em uso de TARV?"

Tipo e Estudo: Ensaio Clínico prospectivo.

Critérios de Inclusão: ≥ 18 anos, em uso de TARV (inibidores de transcriptase reversa nucleosídeos e não nucleosídeos, inibidor de protease), com alterações lipídicas e glicêmicas e que assinem o Termo de Consentimento Livre e Esclarecido (TCLE).

Critérios de Exclusão: gestantes; indígenas, doença oportunista ativa; deficiência mental; uso de drogas ilícitas.

Os participantes serão selecionados para o estudo durante 04 meses (de janeiro a abril de 2011).

Os participantes incluídos no estudo serão aqueles com diagnóstico de HIV/AIDS em uso de TARV, atendidos nos Hospitais Dia de referência na cidade de Campo Grande/MS (Profª Esterina Corsini do Núcleo de Hospital Universitário (NHU) da Universidade Federal de Mato Grosso do Sul (UFMS) e do Centro de Doenças Infecto-Parasitárias (CEDIP) do Serviço Ambulatorial Especializado (SAE) da Secretaria Municipal de Saúde Pública de Campo Grande, MS.

Duração do estudo: 12 meses, sendo que cada participante será acompanhado no mínimo durante 03 meses.

Serão formados 02 grupos envolvendo 80 participantes de ambos os sexos em uso de TARV em cada grupo, totalizando 160 participantes.

Grupo I receberá:

- avaliação nutricional por antropometria,
- avaliação do consumo alimentar,
- orientação nutricional sobre alimentação saudável.

Grupo II receberá:

- avaliação nutricional por antropometria,
- avaliação do consumo alimentar,
- orientação nutricional sobre alimentação saudável e,
- composto bioativo para consumo diário entregue nas consultas de nutrição para consumo durante 90 dias.

Os alimentos presentes no composto bioativo (semente de linhaça, aveia e proteína texturizada de soja) serão pesados e envasados em embalagem plástica atóxica e estéril no Serviço de Nutrição e Dietética do Núcleo de Hospital Universitário e avaliados

quanto a sua inocuidade no Laboratório de Microbiologia dos Alimentos do Departamento de Tecnologia dos Alimentos e Saúde Pública/Centro de Ciências Biológicas e da Saúde/UFMS.

Todos participantes receberão orientação nutricional. As condutas serão baseadas no National Cholesterol Education Program (NCEP), nas diretrizes da Associação Americana do Coração (AHA), da Associação Americana de Diabetes (ADA), do Guia Alimentar para a População Brasileira e da Associação Americana de Dietética para indivíduos infectados com HIV/Aids.

As avaliações antropométricas (IMC, circunferência cintura-quadril, circunferência abdominal) e do consumo alimentar, por meio do método quantitativo recordatório de 24 h, serão realizadas nas consultas de nutrição. As avaliações antropométricas e do consumo alimentar e a aplicação do questionário estruturado serão realizadas pelos pesquisadores mediante treinamento, com vistas à obtenção de uniformidade na abordagem e análises dos resultados.

A avaliação bioquímica ocorrerá conforme rotina médica de solicitação de exames dos Hospitais Dia realizados nos respectivos laboratórios de análises clínicas. A coleta de sangue para as análises de perfil lipídico, triglicerídeos, glicemia e insulina de jejum serão realizadas por técnicos treinados, funcionários dos laboratórios das unidades (Hospital Dia do HU/UFMS e Hospital Dia do CDIP/SESAU).

Será elaborado um QUESTIONÁRIO ESTRUTURADO contendo as variáveis do estudo: aspectos sociodemográficos, uso de tabaco e álcool, prática de atividade física, história de doenças dislipidêmicas familiar, dados antropométricos (Peso, Estatura, Razão Circunferência Cintura-Quadril, Circunferência Abdominal), e consumo

alimentar (história alimentar, recordatório de 24 horas, e freqüência alimentar), resultados dos exames laboratoriais.

Os dados serão armazenados em planilha do Microsoft Excell, analisados em testes estatísticos para descrever os resultados e estabelecer as possíveis correlações entre as variáveis.

4. OBJETIVOS GERAIS E ESPECÍFICOS

Objetivo Geral

Avaliar a influência da intervenção nutricional por meio de composto bioativo no controle da dislipidemia e hiperglicemia em indivíduos HIV positivo que utilizam terapia antiretroviral (TARV).

Objetivos Específicos

- Realizar orientação sobre alimentação saudável;
- Comparar as necessidades calóricas com o consumo alimentar;
- Realizar avaliação nutricional;
- Analisar os resultados bioquímicos;
- Relacionar os resultados laboratoriais com a utilização do composto bioativo.

5. LINHA TEMÁTICA

Área de Conhecimento: Ciências da Saúde

LINHA TEMÁTICA: Promoção da Saúde

Subtema: AIDS.

6. PROJETO APROVADO EM AGÊNCIA DE FOMENTO

* Fundação de Apoio no Desenvolvimento do Ensino, Ciência e Tecnologia do Estado de Mato Grosso do Sul – Edital FUNDECT/MS/CNPq /SES Nº 07/2009 – Saúde. Protocolo:13927.282.4892.27082009. Termo de Outorga: 0025/10

7. RESULTADOS, PRODUTOS, AVANÇOS E APLICAÇÕES ESPERADAS

* Controle e redução dos níveis séricos de colesterol total e LDL-c, triglicerídeos e glicose, viabilizando melhor tratamento dos efeitos advindos do uso da TARV.

***** Caracterizar a interdisciplinaridade com ações interprofissionais e interinstitucionais.

* Gerar produção científica como artigos e tese de doutorado.

8. Fontes de Financiamento: contrapartida institucional e instituições colaboradoras/parcerias

- Fundação de Apoio ao Desenvolvimento do Ensino, Ciência e Tecnologia do Estado de Mato Grosso do Sul (FUNDECT).
- Universidade Federal de Mato Grosso do Sul (UFMS).
- Secretaria Municipal de Saúde Pública - Campo Grande-MS (SESAU).

9. REFERÊNCIAS BIBLIOGRÁFICAS

1 - BRASIL. Recomendações para terapia antiretroviral em adultos e adolescentes infectados pelo HIV. Secretaria de Vigilância em Saúde Programa Nacional de DST e Aids. Ministério da Saúde. Brasília. 2007; 5-6; 85-90.

2 – Gotlieb, S.L.D, Castilho, E.A, Buchalla, C.M. O Impacto da Aids na esperança de vida. Brasil, 2000. Boletim epidemiológico Aids. Coordenação Nacional de DST e Aids. Ano XVI, n.1. Ministério da Saúde do Brasil. Brasilía: 2002.

3 – Marins, J.R.P, Jamal, L.F, CHEN, S, *et al*. Sobrevivência atual dos pacientes com Aids no Brasil. Evidência dos resultados de um esforço nacional. Boletim epidemiológico Aids. Coordenação Nacional DST e Aids. Ministério da Saúde do Brasil. Ano XV. Brasília: n.2. 2002

4 –DOMINGUES, H. Efeitos Metabólicos Associados à Terapia Anti-retroviral Potente em Pacientes com AIDS em Campo Grande, MS [dissertação]: Programa Multiinstitucional de Pós-Graduação em Ciências da Saúde- Convênio Rede Centro-Oeste UnB/UFG/UFMS. 2006.

5 – DOURADO, I; VERAS, M.A.S.M, BARREIRA, D; BRITO, A;M. Aids epidemic trends after the introduction of antireroviral therapy in Brazil. Rev. Saude Publica. 2006; 40(Suppl):9-17.

6 – CAROSI, G.; QUIROS-ROLDAN, E.; TORTI. C.; ANTINORI, A.; BEVILACQUA, M.; BONADONNA, R.C.; et al. First Italian Consensus Statemant on Diagnosis, Prevention anda treatment of Cardivascular Complications in HIV-infected pacients in the HAART era (2006). Infection. 2007;35(3):134-42.

7 - STEIN,J.H. Managing Cardiovascular Risk in Pacients with HIV infection. J. Acquir Immne Defic Syndr. 2005;38(2):115-23.

8 - SOUZA, P. H. M.; SOUZA NETO, M. H.; MAIA, G. A. Componentes funcionais nos alimentos. Boletim da SBCTA. 2003;37(2)127-135.

9 - CANDIDO, L.M.B.; CAMPOS, A. M. Alimentos funcionais. Uma revisão. Boletim da SBCTA. 2005;29(2)193-203.

10 -S**.** Dodin, A. Lemay, H. Jacques, F. Légaré, J.-C. Forest and B. Mâsse. The Effects of Flaxseed Dietary Supplement on Lipid Profile, Bone Mineral Density, and Symptoms in Menopausal Women: A Randomized, Double-Blind, Wheat Germ Placebo-Controlled Clinical Trial. The Journal of Clinical Endocrinology & Metabolism. 2006;90(3):1390-1397.

11 – American Dietetic Association . Position of the American Dietetic Association and Dietitians of Canada: Nutrition intervention in the care of persons with human immunodeficiency vírus infection. J Am Diet Assoc. 2004;104:1425-41.

12 - Prim C.R; Précoma D.B. Papel da semente de linhaça na anti – aterogênese e na ação antiinflamatória em coelhos submetidos à dieta hipercolesterolêmica – Análise imunohistológica.

http://www.agenciapuc.pucpr.br/agencia/projetos/ver2.aspx?codigo_projeto=113 acesso em 19/08/2009. Dissertação de mestrado em Ciências da Saúde (Conceito CAPES 5). Ano de Obtenção: 2008 pela Pontifícia Universidade Católica do Paraná, PUC-PR, Brasil.

13 - Boletim Epidemiológico DST/Aids. Programa Nacional DST/AIDS. Ministério da Saúde. 2008.
