# Supplementary material for: Effectiveness of a bioactive food compound in anthropometric measures of individuals with HIV/AIDS: A nonrandomized trial
Source: PLoS One. 2018 Feb 9;13(2):e0191259. doi: 10.1371/journal.pone.0191259 (PMC5806863; doi:10.1371/journal.pone.0191259)
Supplement: S6 File — (PDF) [file pone.0191259.s006.pdf]

BIOATIVE COMPOUND: NUTRITIONAL THERAPEUTICS IN LIPIDIC AND GLYCEMIC ALTERATIONS FOR HIV INFECTION IN INDIVIDUALS UNDER COMBINED ANTIRETROVIRAL THERAPY.

ROSÂNGELA DOS SANTOS FERREIRA

E-mail: rosangela.ferreira@ufms.br

Coordinator

Universidade Federal de Mato Grosso do Sul

2009

Campo Grande-MS

1. LITERATURE REVIEW

In Latin America, Brazil was the first country to adopt the policy of universal distribution of drug therapy for population infected by HIV/AIDS. Since 1991, antiretroviral drugs have been universally distributed, and since 1996, after the 11th International AIDS Conference held in Vancouver, there has been a historic milestone and the Brazilian program has had the free, mandatory and universal policy of distribution of antiretroviral therapy (ART), guaranteed by Law 9,313 of November 13, 1996(1).

Thereafter, there was a progressive change in the morbidity and mortality profile by HIV infection, demonstrated through reduced hospital admissions, reduced occurrence of associated opportunistic infections and consequently, deaths secondary to AIDS. The disease had a better prognosis; patients' survival increased, HIV infection came to be considered as a disease of evolutionary and chronic character and potentially controllable (2,3).

High-potency ART is in the combination of three classes of antiretroviral drugs associated with two Nucleoside Reverse Transcriptase Inhibitors (NRTIs) with a Protease Inhibitor (PI) or a Non-Nucleoside Reverse Transcriptase Inhibitor (NNRTI), showing control of infection evolution, with better prognosis of life of these individuals (4). On the other hand, adverse events associated with therapy are evidenced by the development of lipodystrophy syndrome, characterized by dyslipidemia, hypercholesterolemia and hypertriglyceridemia, glycemic alterations and morphological changes with lipoatrophy and lipohypertrophy (5).

Studies show side effects of drugs on lipid and glycemic changes. Domingues (4) observed hypercholesterolemia in 68.0%, 57.1%, 45.6% and 32.6% of patients taking indinavir/ritonavir, saquinavir/ritonavir, nelfinavir and lopinavir/ritonavir, respectively.

In this scenario, nutritional intervention had positive impact on the prevention and primary control of changes in the lipid and glycemic profile of individuals (6,7).

Seeking to combine food strategies to coadjuvant treatment for side effects of high-potency ART, we proposed to use special-purpose foods, as functional foods for their bioactive properties, besides basic nutrition. They will be consumed in conventional daily diets, showing ability to regulate body functions and bringing specific physiological benefits to help protect against cardiovascular diseases (CAD) and diabetes mellitus (8,9). Thus, the interest in studying this feeding protocol through a bioactive compound in HIV-infected individuals is reinforced.

We intend to introduce functional foods as part of healthy eating to these individuals, aiming at control and adjuvant therapy in the development of CAD and glycemic change due to the use of ART.

This study will deal with the development of a bioactive compound consisting of: **Flaxseed** (*Linum usitatissimum)* contains substances such as lignan, fibers and linolenic fatty acid which have cardioprotective effects (10) with a therapeutic dosage of 10 g (01 DSL), suitable for atherosclerosis model, suitable to act as preventive and therapeutic in the regression of atherosclerosis and consequent cardiovascular diseases; **oat** (*Avena sativa*) in coarse flakes, represented by bran and oats, because it has higher dietary source of B-glucan, in the amount of 20 g (1 tbsp), suitable for maintenance and adjustment of glycemia within normality levels; **texturized soy protein** (*Glycine max*) in the dosage of 10 g (02 tbsp), protein with low content of saturated lipid and cholesterol in chemical composition, with hypocholesterolemic property, it decreases platelet aggregation and atherosclerotic lesions. These foods have cardioprotective effects and glycemic adequacy (11).

The established amounts will be considered in this study as test dosages for proposed purposes, namely: reduction of total cholesterol (TC), high density lipoprotein (LDL-c), triglycerides (TG) and blood glucose.

Key words: HIV, AIDS, dyslipidemias, hyperglycemia, functional foods.

2. BACKGROUND AND JUSTIFICATION

Brazil has been undergoing a process of reversal of mortality curves known as the epidemiological transition, in which there is a decline in mortality from infectious diseases and a concomitant increase in mortality from non-communicable chronic diseases (2).

Currently, HIV/AIDS epidemic is considered stable in the country, assuming a chronic disease character, due to high-potency antiretroviral therapy (ART). Studies show about two thousand adults diagnosed with HIV/AIDS between 1998 and 1999, more than half (60%) remained alive for at least 108 months after diagnosis, confirming survival growth of Brazilian patients. A historical series from 1980 to June 2008 registered 506,499 cases of AIDS in Brazil. During these years, 205,409 deaths occurred as a result of illness (2,3).

The Southeast Region has the highest percentage of notifications, 60.4% or 305,725 cases. The South concentrates 95,552 (18.9%), the Northeast 58.3481 (15%), the Midwest 28,719 (5.7%) and the North 18,155 (3.6%) (13).

Early diagnosis followed by access to antiretroviral drugs and adequate clinical follow-up may increase patient survival. The use of ART has positive impact on life after diagnosis in patients diagnosed as still asymptomatic, having longer survival than those who have already developed opportunistic diseases. Patients with higher education (high school and undergraduate) live longer; women have greater survival; those who

are sexually infected live longer than those exposed to the virus for use of injectable drugs; those who do prophylaxis of pneumocystosis (potentially severe pneumonia) also live longer (1).

Countries such as Brazil, which opted for universal access to treatment in the 1990s, determined change in the disease natural history. In addition to the universal offer of high-potency ART, structuring the health system was fundamental to improve quality of life of people living with HIV/AIDS (1). However, it is still necessary to strengthen the response of health services to the adverse effects of treatment by encouraging a healthy lifestyle (adequate diet and physical activity), preventing the occurrence of cardiovascular diseases and lipodystrophy (6). Functional clinical nutrition is a contemporary way of approaching the science of nutrition to evaluate the organism-food interaction and nutrition process. In this way, functional foods are capable of acting in the metabolism and human physiology, promoting beneficial effects to the health, being able to delay chronic and/or degenerative diseases and to improve the quality and life expectancy of people. Functional nutrition takes into account the importance of physiological and functional integrity of gastrointestinal tract (9).

Thus, interference with eating habits and processes is important to effectively interfere with ART side effects.

Based on this context, functional nutrition emerges with some principles to assure a better supply of substrates to the organism, determining a new era in the control of lipid and glycemic changes of individuals submitted to ART.

Therefore, we justify the search for nutritional strategies aiming at preventing and participating in the control of adversities resulting from antiretroviral drugs in individuals infected with HIV/AIDS.

3. METHODS

This study was conceived by the following hypothesis: "Is there a relation between a diet with functional activity (bioactive compound), dyslipidemias and glycemic alteration in HIV-infected individuals under use of ART?"

Type and Study: Prospective Clinical Trial.

Inclusion Criteria: age ≥ 18 years, under ART (nucleoside and non-nucleoside reverse transcriptase inhibitors, protease inhibitor), with lipid and glycemic alterations and who had signed the Free and Informed Consent Term (FICT).

Exclusion Criteria: pregnant women; Indigenous, active opportunistic disease; mental disability; use of illicit drugs.

Participants will be selected during 4 months (January to April 2011).

Participants included in the study will be those diagnosed with HIV/AIDS under ART, attended at the Day Hospital of reference in the city of Campo Grande/MS (Prof. Esterina Corsini of the University Hospital Nucleus (NHU) at the Universidade Federal de Mato Grosso do Sul (UFMS) and the Infectious-Parasitic Diseases Center (IPDC) of the Specialized Ambulatory Service (SAS) of the Municipal Department of Public Health of Campo Grande, MS.

Duration: 12 months. Each participant will be followed for at least 03 months.

Two groups will be formed involving 80 participants of both sexes under ART in each group, totaling 160 participants.

Group I will receive:

- nutritional assessment by anthropometry,

- assessment of food consumption,
- nutritional guidance on healthy eating.

Group II will receive:

- nutritional assessment by anthropometry,
- assessment of food consumption,
- nutritional guidance on healthy eating and,
- bioactive compound for daily consumption delivered in nutrition consultations for 90 days.

Foods present in the bioactive compound (flaxseed, oats and texturized soy protein) will be weighed and packaged in non-toxic and sterile plastic packaging at the Nutrition and Dietetics Department of the University Hospital Nucleus and evaluated for their safety in the Laboratory of Microbiology of Food from the Department of Food Technology and Public Health/Center of Biological and Health Sciences/UFMS.

All participants will receive nutritional counseling. The guidelines will be based on the National Cholesterol Education Program (NCEP), the American Heart Association (AHA), the American Diabetes Association (ADA), and the Food Guide for Brazilian Population and the American Dietetic Association Guidelines for individuals infected with HIV/Aids.

Anthropometric assessments (BMI, waist-hip circumference, waist circumference) and food consumption using the 24-hour recall quantitative method will be performed in nutrition consultations. Anthropometric and food consumption assessments and the

application of structured questionnaire will be carried out by trained researchers aiming at obtaining uniformity in the approach and analysis of results.

Biochemical evaluation will occur according to the medical routine of requesting exams to Day Hospitals performed in the proper laboratories for clinical analysis. Blood collection for analysis of lipid profile, triglycerides, glycemia and fasting insulin will be performed by trained technicians, employees of the units' laboratories (Day Hospital of UH/UFMS and Day Hospital of IPDC/SAS).

A STRUCTURED QUESTIONNAIRE containing study variables: sociodemographic aspects, tobacco and alcohol use, physical activity practice, history of familial dyslipidemic diseases, anthropometric data (Weight, Height, Waist-Hip Circumference, Abdominal Circumference), and food consumption (Food history, 24-hour recall, and food frequency), laboratory test results.

Data will be stored in Microsoft Excel worksheet and analyzed through statistical tests to describe results and to establish the possible correlations between variables.

4. GENERAL AND SPECIFIC OBJECTIVES

General objective

Evaluate the influence of nutritional intervention by bioactive compound in the control of dyslipidemia and hyperglycemia in HIV-positive individuals under antiretroviral therapy (ART).

Specific objectives

- Conduct guidance on healthy eating;
- Compare caloric needs with food consumption;

- Perform nutritional assessment;
- Analyze biochemical results;
- Relate laboratory results to the use of the bioactive compound.

5. THEMATIC LINE

Area of Knowledge: Health Sciences

THEMATIC LINE: Health promotion

Subtheme: AIDS.

6. PROJECT APPROVED BY A FOMENTATION AGENCY

* Fundação de Apoio no Desenvolvimento do Ensino, Ciência e Tecnologia do Estado de Mato Grosso do Sul – Edital FUNDECT/MS/CNPq /SES Nº 07/2009 – Saúde. Protocol:13927.282.4892.27082009. Term of Grant: 0025/10

7. EXPECTED RESULTS, PRODUCTS, ADVANCES AND APPLICATIONS

* Control and reduction of serum levels of total cholesterol and LDL-C, triglycerides and glucose, allowing better treatment of effects arising from ART.

* Characterize interdisciplinary with interprofessional and interinstitutional actions.

* Generate scientific output as articles and doctoral thesis.

8. Sources of Funding: Institutional counterpart and collaborating/partnerships institutions

- Fundação de Apoio ao Desenvolvimento do Ensino, Ciência e Tecnologia do Estado de Mato Grosso do Sul (FUNDECT).
- Universidade Federal de Mato Grosso do Sul (UFMS).
- Secretaria Municipal de Saúde Pública - Campo Grande-MS (SESAU).

9. REFERENCES

1 - BRASIL. Recomendações para terapia antiretroviral em adultos e adolescentes infectados pelo HIV. Secretaria de Vigilância em Saúde Programa Nacional de DST e Aids. Ministério da Saúde. Brasília. 2007; 5-6; 85-90.

2 – Gotlieb, S.L.D, Castilho, E.A, Buchalla, C.M. O Impacto da Aids na esperança de vida. Brasil, 2000. Boletim epidemiológico Aids. Coordenação Nacional de DST e Aids. Ano XVI, n.1. Ministério da Saúde do Brasil. Brasilía: 2002.

3 – Marins, J.R.P, Jamal, L.F, CHEN, S, *et al.* Sobrevivência atual dos pacientes com Aids no Brasil. Evidência dos resultados de um esforço nacional. Boletim epidemiológico Aids. Coordenação Nacional DST e Aids. Ministério da Saúde do Brasil. Ano XV. Brasília: n.2. 2002

4 –DOMINGUES, H. Efeitos Metabólicos Associados à Terapia Anti-retroviral Potente em Pacientes com AIDS em Campo Grande, MS [dissertação]: Programa Multiinstitucional de Pós-Graduação em Ciências da Saúde- Convênio Rede Centro-Oeste UnB/UFG/UFMS. 2006.

5 – DOURADO, I; VERAS, M.A.S.M, BARREIRA, D; BRITO, A;M. Aids epidemic trends after the introduction of antireroviral therapy in Brazil. Rev. Saude Publica. 2006; 40(Suppl):9-17.

6 – CAROSI, G.; QUIROS-ROLDAN, E.; TORTI. C.; ANTINORI, A.; BEVILACQUA, M.; BONADONNA, R.C.; et al. First Italian Consensus Statemant on Diagnosis, Prevention anda treatment of Cardivascular Complications in HIV-infected pacients in the HAART era (2006). Infection. 2007;35(3):134-42.

7 - STEIN,J.H. Managing Cardiovascular Risk in Pacients with HIV infection. J. Acquir Immne Defic Syndr. 2005;38(2):115-23.

8 - SOUZA, P. H. M.; SOUZA NETO, M. H.; MAIA, G. A. Componentes funcionais nos alimentos. Boletim da SBCTA. 2003;37(2)127-135.

9 - CANDIDO, L.M.B.; CAMPOS, A. M. Alimentos funcionais. Uma revisão. Boletim da SBCTA. 2005;29(2)193-203.

10 -S**.** Dodin, A. Lemay, H. Jacques, F. Légaré, J.-C. Forest and B. Mâsse. The Effects of Flaxseed Dietary Supplement on Lipid Profile, Bone Mineral Density, and Symptoms in Menopausal Women: A Randomized, Double-Blind, Wheat Germ Placebo-Controlled Clinical Trial. The Journal of Clinical Endocrinology & Metabolism. 2006;90(3):1390-1397.

11 – American Dietetic Association . Position of the American Dietetic Association and Dietitians of Canada: Nutrition intervention in the care of persons with human immunodeficiency vírus infection. J Am Diet Assoc. 2004;104:1425-41.

12 - Prim C.R; Précoma D.B. Papel da semente de linhaça na anti – aterogênese e na ação antiinflamatória em coelhos submetidos à dieta hipercolesterolêmica – Análise imunohistológica.

http://www.agenciapuc.pucpr.br/agencia/projetos/ver2.aspx?codigo_projeto=113 acesso em 19/08/2009. Dissertação de mestrado em Ciências da Saúde (Conceito CAPES 5). Ano de Obtenção: 2008 pela Pontifícia Universidade Católica do Paraná, PUC-PR, Brasil.

13 - Boletim Epidemiológico DST/Aids. Programa Nacional DST/AIDS. Ministério da Saúde. 2008.
