# Supplementary material for: Effectiveness of a bioactive food compound in anthropometric measures of individuals with HIV/AIDS: A nonrandomized trial
Source: PLoS One. 2018 Feb 9;13(2):e0191259. doi: 10.1371/journal.pone.0191259 (PMC5806863; doi:10.1371/journal.pone.0191259)
Supplement: S8 File — (PDF) [file pone.0191259.s008.pdf]

Brazilian Registry of Clinical Trials – Ministry of Health, Brazil

Trial url:http://www.ensaiosclinicos.gov.br/rg/RBR-6m2fch/

Register Number: RBR-6m2fch
